# Supplementary material for: Effectiveness and Safety of Ayurvedic Medicines in Type 2 Diabetes Mellitus Management: A Systematic Review and Meta-Analysis
Source: Front Pharmacol. 2022 Jun 8;13:821810. doi: 10.3389/fphar.2022.821810 (PMC9213670; doi:10.3389/fphar.2022.821810)
Supplement: Supplementary file 1 [file DataSheet1.zip › Supplementary Material/Forest and Funnel Plots/Syzygium cumini/HDL-C.pdf]

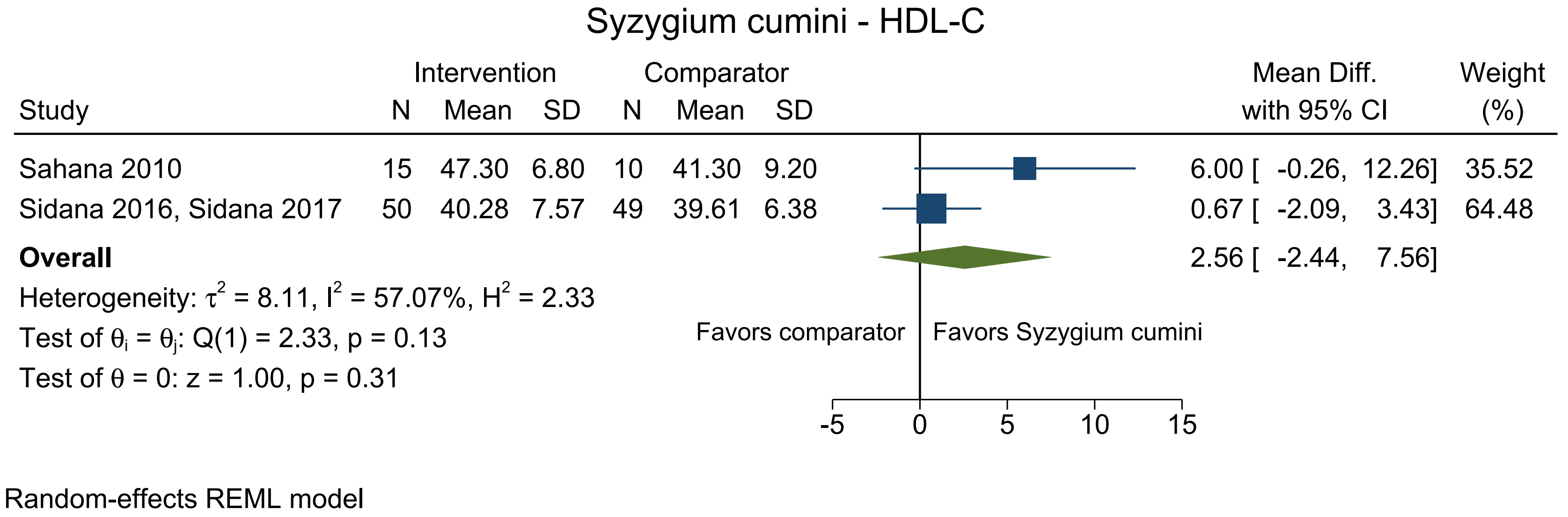
Syzygium cumini - HDL-C
Intervention
Comparator
Study
N
Mean
SD
N
Mean
SD
Mean Diff. with 95% CI
Weight (%)
Sahana 2010 15 47.30 6.80 10 41.30 9.20 6.00 [-0.26, 12.26] 35.52
Sidana 2016, Sidana 2017 50 40.28 7.57 49 39.61 6.38 0.67 [-2.09, 3.43] 64.48
Overall 2.56 [-2.44, 7.56]
Heterogeneity: τ² = 8.11, I² = 57.07%, H² = 2.33
Test of θi = θj: Q(1) = 2.33, p = 0.13
Test of θ = 0: z = 1.00, p = 0.31
Favors comparator
Favors Syzygium cumini
-5
0
5
10
15
Random-effects REML model
